# New Promising Steroidal Aromatase Inhibitors with Multi-Target Action on Estrogen and Androgen Receptors for Breast Cancer Treatment

**DOI:** 10.3390/cancers17020165

**Published:** 2025-01-07

**Authors:** Cristina Amaral, Cristina F. Almeida, Maria João Valente, Carla L. Varela, Saul C. Costa, Fernanda M. F. Roleira, Elisiário Tavares-da-Silva, Anne Marie Vinggaard, Natércia Teixeira, Georgina Correia-da-Silva

**Affiliations:** 1 UCIBIO—Applied Molecular Biosciences Unit, Laboratory of Biochemistry, Department of Biological Sciences, Faculty of Pharmacy, University of Porto, Rua Jorge Viterbo Ferreira, n° 228, 4050-313 Porto, Portugal; cristina-almeida96@hotmail.com (C.F.A.); natercia@ff.up.pt (N.T.); 2 Associate Laboratory i4HB, Institute for Health and Bioeconomy, Faculty of Pharmacy, University of Porto, Rua Jorge Viterbo Ferreira, n° 228, 4050-313 Porto, Portugal; 3 National Food Institute, Technical University of Denmark, 2800 Kongens Lyngby, Denmark; mjopo@food.dtu.dk (M.J.V.); annv@food.dtu.dk (A.M.V.); 4 Univ Coimbra, CERES, Coimbra, Portugal; Univ Coimbra, Coimbra Institute for Clinical and Biomedical Research (iCBR), Clinic Academic Center of Coimbra (CACC), Coimbra, Portugal; Univ Coimbra, Center for Innovative Biomedicine and Biotechnology (CIBB), Azinhaga de Santa Comba, Pólo III, Pólo das Ciências da Saúde, 3000-548 Coimbra, Portugal; carlalvarela@gmail.com; 5 Univ Coimbra, Faculty of Pharmacy, Laboratory of Pharmaceutical Chemistry, Azinhaga de Santa Comba, Pólo III, Pólo das Ciências da Saúde, 3000-548 Coimbra, Portugal; saul@ff.uc.pt; 6 Univ Coimbra, CERES, Faculty of Pharmacy, Laboratory of Pharmaceutical Chemistry, Azinhaga de Santa Comba, Pólo III, Pólo das Ciências da Saúde, 3000-548 Coimbra, Portugaletavares@ff.uc.pt (E.T.-d.-S.)

**Keywords:** breast cancer, endocrine therapy, endocrine resistance, multi-target drugs, polypharmacology, anti-cancer drugs, aromatase inhibitors, aromatase, androgen receptor, estrogen receptor

## Abstract

**Background/Objectives:** Endocrine therapies that comprise anti-estrogens and aromatase inhibitors (AIs) are the standard treatment for estrogen receptor-positive (ER+) (Luminal A) breast cancer—the most prevalent subtype. However, the emergence of resistance restricts their success by causing tumor relapse and re-growth, which demands a switch towards other therapeutic approaches in order to minimize or overcome resistance. Indeed, this clinical limitation highlights the search for new molecules to improve cancer treatment. Recently, strategies that address multiple targets have been emerging, and multi-target drugs have the potential to become the future anti-cancer molecules. Our group has been searching for new multi-target compounds, and as part of this, our study aims to understand the anti-cancer and multi-target potential of three new steroidal aromatase inhibitors (AIs): 7α-methylandrost-4-en-17-one (**6**), 7α-methylandrost-4-ene-3,17-dione (**10a**) and androsta-4,9(11)-diene-3,17-dione (**13**). **Methods:** Their in vitro actions and molecular mechanisms were elucidated in a sensitive ER+ aromatase-overexpressing breast cancer cell line, MCF-7aro cells, as well as in an AI-resistant ER+ breast cancer cell line, LTEDaro cells. **Results:** All the new AIs (10 µM) prevented the proliferation of MCF-7aro cells by arresting cell cycle progression. Interestingly, all AIs (10 µM) act as androgen receptor (AR) agonists and modulate ER levels, synthesis and signaling to induce the apoptosis of ER+ breast cancer cells. Additionally, these new AIs (10 µM) also re-sensitize resistant cells by promoting apoptosis, offering a therapeutic benefit. **Conclusions:** Overall, new steroidal polypharmacological compounds have been discovered that, by acting as AIs, ER modulators and AR agonists, impair ER+ breast cancer cell growth. Overall, this study is a breakthrough on drug discovery as it presents new molecules with appealing anti-cancer properties and multi-target action for the treatment of ER+ breast cancer.

## Simple Summary

The occurrence of resistance to the standard treatment for estrogen receptor-positive (ER+) (Luminal A) breast cancer emphasizes the search for new molecules. In recent years, the interest in multi-target drugs has been growing, and they have even been identified as future therapeutic strategies. In line with this, this study aims to understand the anti-cancer action and multi-target potential of three new steroidal aromatase inhibitors (AIs): 7α-methylandrost-4-en-17-one (**6**), 7α-methylandrost-4-ene-3,17-dione (**10a**) and androsta-4,9(11)-diene-3,17-dione (**13**). We report new compounds that are able to impair the growth of sensitive and resistant ER+ breast cancer cells by acting as AIs, ER modulators and AR agonists, which is a therapeutic advantage over the current drugs applied in clinic. Therefore, this study is a breakthrough on drug discovery as it allows the discovery of new multi-target drugs for the treatment of ER+ breast cancer.

## 1. Introduction

Endocrine therapy that comprises the third generation of aromatase inhibitors (AIs)—Exemestane (Exe), Anastrozole (Ana) and Letrozole (Let)—as well as the selective estrogen receptor modulator (SERM) Tamoxifen and the selective estrogen receptor degrader (SERD) Fulvestrant have been used as the first-line therapy for both post- and pre-menopausal women with estrogen receptor-positive (ER+) breast cancer in early and advanced stages [1,2,3]. In fact, most of the cases (around 70–85%) are ER+ [1,2,4], with endocrine therapy being the main treatment option. Despite the success of these therapies, clinical evidence suggests that in the adjuvant setting, approximately 50% of patients may relapse [5] due to the occurrence of endocrine resistance. To minimize or overcome this worrying adverse effect, several therapeutic strategies, like combinations of endocrine therapy with PI3K, AKT mTOR, or CDK4/6 inhibitors, are being applied. Nonetheless, these combined strategies either do not improve overall survival or induce treatment-related toxicity [2,3,6,7,8], which often leads to resistance maintenance. In fact, breast cancer cases showing resistance to CDK4/6 inhibitors are emerging [8,9,10,11,12,13] and a new approach with SERDs is gaining clinical attention, as evidenced by the ongoing clinical trials involving oral SERDs and the already-approved Elacestrant [8,9,10,14].

**Figure 1:**
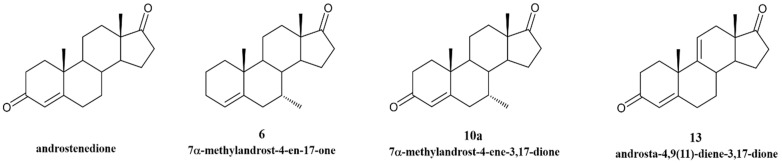
Chemical structure of the compounds 7α-methylandrost-4-en-17-one (**6**), 7α-methylandrost-4-ene-3,17-dione (**10a**) and androsta-4,9(11)-diene-3,17-dione (**13**).

Traditionally, anti-cancer drugs have been designed to target a single biological entity based on the idea of “one disease—one target—one drug” [15,16,17,18]. On the other hand, recent new treatment strategies involve the design and synthesis of compounds as multi-targeted anti-cancer agents. Thus, cancer treatment may involve either the combination of multiple drug treatments or the application of polypharmacology (also called the multi-targeting approach). The latter is focused on the concept of “one drug-multiple targets”, where a single molecule is designed to simultaneously modulate two or more molecular targets [15,16,17,18,19]. In fact, the complexity of cancer, with the recruitment of key proteins and multiple signal transduction pathways linked to the promotion and progression of the disease and the clinical limitations of endocrine therapy, has highlighted the drawbacks of single-target drugs [1,19,20]. The capacity of a drug to simultaneously target the enzyme aromatase, the ERα and/or the androgen receptor (AR) is clinically important, as these are three fundamental therapeutic targets in ER+ tumors [1,3]. In fact, ERα is associated with cell growth and survival, while aromatase is responsible for a key step in the biosynthesis of estrogens and, hence, the growth of estrogen-dependent cells [1,2,3,21]. In addition, the AR is a steroid hormone receptor widely detected in breast cancer, and approximately 80–95% of all ER+ breast cancers overexpress this receptor [22,23]. Evidence suggests that the AR might act as a tumor suppressor in ER+ breast cancer or a tumor promoter in ER- tumors. Thus, modulating AR activity could be a potential treatment strategy [1,2,3,21,24,25,26,27]. Moreover, as the active binding sites of ER and aromatase present similar sizes and contain equivalent residues, suggesting the binding of the same ligands [1], molecules that present dual anti-estrogen and anti-aromatase features are posed as future drugs for ER+ breast cancer therapy [28,29]. Thus, when single-target molecules are unsuccessful or present severe clinical limitations, drugs with multi-target action arise as promising and effective strategies [1,20]. Indeed, multi-target drugs may possess superior pharmacokinetics and efficacy, a lower probability of resistance and a better safety profile correlated with a lower probability of drug interactions when compared to single-target drugs or even combination therapy [1,15,16,17,18,20]. In fact, additive therapeutic effects may be achieved when the targets belong to functionally complementary pathways or to the same pathway [15,17,19]. In addition to our group [28,30,31,32,33,34,35], other teams [29,36,37,38] have been investigating multi-target or polypharmacological drugs for ER+ breast cancer. Recently, we synthesized three new potent AIs: 7α-methylandrost-4-en-17-one (**6**), 7α-methylandrost-4-ene-3,17-dione (**10a**) and androsta-4,9(11)-diene-3,17-dione (**13**) [39,40]. These molecules were designed based on the steroidal scaffold of androstenedione, the natural substrate of aromatase, with different chemical modifications in A- and B-rings (compound **6**), or only in the B-ring (compound **10a**) or in the C-ring (compound **13**) (Figure 1). We already demonstrated that these molecules presented an anti-aromatase activity in human placental microsomes of 97.39%, 94.35% and 95.22% for compound **6**, **10a** [40] and **13 [39]**, respectively, as well as an IC_50_ value of aromatase inhibition of 0.405 µM, 0.27 µM and 0.25 µM, respectively. Moreover, these new AIs also present an anti-aromatase activity above 90% in ER+ breast cancer cells that overexpress aromatase (MCF-7aro cells) [39,40]; thus, they are being considered as potent steroidal AIs.

In this study, our goal is to unveil the anti-cancer activities of these three steroidal AIs in sensitive (MCF-7aro) and resistant (LTEDaro) ER+ breast cancer cells, cell models that mimic the Luminal A breast cancer subtype, as well as their multi-target potential.

## 2. Material and Methods

### 2.1. New Steroidal Aromatase Inhibitors Under Study

The anti-cancer potential of the new potent AIs that our group has previously synthesized [39,40]—the steroids 7α-methylandrost-4-en-17-one (**6**), 7α-methylandrost-4-ene-3,17-dione (**10a**) and androsta-4,9(11)-diene-3,17-dione (**13**) (Figure 1)—was explored in non-tumor and in ER+ sensitive and resistant breast cancer cells.

### 2.2. Cell Cultures

Two different non-tumor cell lines—the human foreskin fibroblast cell line (HFF-1) (RRID:CVCL_3285) (SCRC-1041, American Type Culture Collection (ATCC), Manassas, VA, USA) and the human non-tumorigenic epithelial breast cell line (MCF-10A) (RRID:CVCL_0598) (CRL-10317, American Type Culture Collection (ATCC), Manassas, VA, USA)—were applied to investigate the cytotoxic effects of the AIs **6**, **10a** and **13** in non-tumor cells. The cell culture conditions of HFF-1 cells are the ones previously described by our group [30,34,41]. MCF-10A cells were regularly maintained in DMEM F/12 with no phenol red culture medium complemented with HuMEC supplement, 5% heat-inactivated horse serum, 2 mmol/L L-glutamine and 1% penicillin-streptomycin-amphotericin B solution (all acquired from Gibco Invitrogen Co., Paisley, Scotland, UK).

To investigate the anti-cancer properties of the AIs **6**, **10a** and **13**, two different ER+ breast cancer aromatase-overexpressing cell lines models, MCF-7aro (sensitive) and LTEDaro (resistant), which are known to mimic the Luminal A breast cancer subtype, were used. MCF-7aro (RRID:CVCL_9580) was derived from the parental ER^+^ breast cancer cell line, MCF-7, as previously reported [42,43]. As these cells overexpress aromatase, they perfectly mimic in vitro the microenvironment of ER+ tumor and are thus considered a suitable cell line model to study this cancer subtype and AIs [44,45,46]. This cell model was maintained under routine culture conditions already published in previous works [25,28,34,47,48]. For the experiments and as proliferation-inducing agents, MCF-7aro cells must be stimulated with 1 nM of testosterone (T) or with 1 nM of estradiol (E_2_) (both from Sigma-Aldrich Co., Saint Louis, MO, USA), which are the substrate and the product of aromatase enzyme, respectively [28,47,48]. The long-term estrogen-deprived human ER^+^ aromatase-overexpressing breast cancer cell model, LTEDaro cells (RRID:CVCL_W348), was generated from the parental MCF-7aro cells after prolonged culture in steroid-depleted medium, in order to mimic in vitro the adaptation to estrogen withdrawal and thus simulate the acquired resistance to the reference AIs applied in the clinic. Thus, this is a suitable cell line to assess the compounds’ behavior in endocrine resistance [25,45,46,49]. LTEDaro cells have been extensively characterized—they overexpress functional aromatase and ER and do not respond to endocrine therapy, as confirmed by a second-line treatment with AIs [45,46,49,50,51,52]. This cell line model was maintained under routine culture conditions, as already described by our group [25,35,53]. Both sensitive and resistant breast cancer cell models were kindly supplied by Professor Shiuan Chen (Beckman Research Institute, City of Hope, Duarte, CA, USA).

The stock solutions of E_2_ and T were prepared in absolute ethanol, while AIs **6**, **10a** and **13**, as well as Exe, Casodex (CDX) and ICI 182,780, were prepared in 100% DMSO (all supplied by Sigma-Aldrich Co., Saint Louis, MO, USA, except the new AIs under study). For the experiments, fresh solutions of all these compounds were prepared in the appropriate culture medium for each cell model. The final concentrations of DMSO and ethanol in the experiments considering the controls and AI-treated cells were lower than 0.01% and 0.05%, respectively. In each independent assay, we used control cells that were not treated with any of the AIs studied.

### 2.3. Studies of Cell Viability

To explore the effects of the AIs **6**, **10a** and **13** on HFF-1, MCF-10A, MCF-7aro and LTEDaro cells, we carried out two methods for estimating cell viability effects: 3-(4,5-dimethylthiazol-2-yl)-2,5-diphenyltetrazolium (MTT) and the release of the enzyme lactate dehydrogenase (LDH) into the culture medium. The cellular densities in 96-well plates were as follows: 7.5 × 10^3^ cells/mL (6 days) for HFF-1 cells; 1 × 10^4^ cell/mL (6 days) for MCF-10A; 2 × 10^4^ cells/mL (3 days) and 1 × 10^4^ cells/mL (6 days) for MCF-7aro and LTEDaro cells. After 24 h of adherence, each cell line was treated for 3 or 6 days with the AIs **6**, **10a** and **13** (1–25 µM). As described above, MCF-7aro cells were additionally incubated with the proliferation-inducing hormones T or E_2_ and to explore the involvement of ER and AR; they were also treated with AIs and the ER degrader ICI 182 780 (100 nM) or the AR antagonist CDX (1 µM).

Following the end of each incubation time, MTT (0.5 mg/mL; Sigma-Aldrich Co., Saint Louis, MO, USA) and LDH release assays were carried out as reported [28]. Data are represented as the relative percentage in relation to the control cell values (100% of cell viability and 1 unit for LDH release assay).

### 2.4. Analysis of Cell Cycle Progression by Flow Cytometry

To estimate the behavior of AIs **6**, **10a** and **13** on cycle progression, it was assessed by flow cytometry. We measured the DNA content of AI-treated (10 µM) and untreated MCF-7aro cells (7 × 10^5^ cells/mL), all stimulated with T (1 nM) and exposed over 3 days to the different compounds. Using the BD Accuri™ C6 cytometer (San Jose, CA, USA), equipped with BD Accuri™ C6 analysis software, the DNA content was evaluated by acquiring at least 40,000 events, as previously described [28,33]. The impact of each AI on cell cycle progression was analyzed, and the results are plotted as a percentage of single-cell events at each cell cycle stage, the G_0_/G_1_, S and G_2_/M phases.

### 2.5. Analysis of Apoptotic Cell Death

To investigate the occurrence of apoptotic cell death on sensitive and resistant cells treated with AIs **6**, **10a** and **13**, the activation of caspase-7 was measured by luminescent assays with a Caspase-Glo^®^ 3/7 kit (Promega Corporation, Madison, WI, USA), as usually performed by our group [28,54]. Both sensitive and resistant cells plated at a cell density of 2 × 10^4^ cells/mL on 96-well white plates were treated with AIs **6**, **10a** and **13** (10 μM) in the presence or absence of CDX (1 µM) or ICI 182 780 (100 nM) over 3 days. Staurosporine (STS, Sigma-Aldrich Co., Saint Louis, MO, USA) at 10 µM was used as a positive control. It should be pointed out that since MCF-7 cells are deficient on caspase-3 [55], and as MCF-7aro and LTEDaro cell lines are generated from these cells [42,43], we only determined the activity of caspase-7 using the Caspase-Glo^®^ 3/7 kit. Luminescence data are expressed as relative luminescence units (RLUs).

### 2.6. Western Blot Assay

The protein expression levels of either ERα or AR in MCF-7aro cells treated with compounds **6**, **10a** and **13** were investigated by Western blot. These cells that were plated in six-well plates (7 × 10^5^ cells/mL) were incubated with T (1 nM) with or without AIs **6**, **10a** and **13** (10 μM) over 3 days. As a positive control for ERα, we used cells treated with T plus ICI 182 780 (100 nM). Western blot experiments were performed as previously reported [25,28]. The mouse monoclonal AR (1:200, sc-7305) and the mouse monoclonal ERα (1:200, sc-8002) (Santa Cruz Biotechnology, Santa Cruz, CA, USA) were used as primary antibodies to study AR and ERα, respectively. A mouse monoclonal anti-β-tubulin antibody (1:500, sc-5274; Santa Cruz Biotechnology, Santa Cruz, CA, USA) was used to control loading variations. As a secondary antibody, we used the peroxidase-conjugated goat anti-mouse antibody (1:2000, G21040; Thermo Fisher, Waltham, MA, USA).

### 2.7. RNA Extraction and qPCR

To investigate the effects of AIs **6**, **10a** and **13** in the transcription of *ESR1*, *EGR3*, *AREG* and *TFF1* genes, we performed qPCR analysis, as previously described [28,48]. *ESR1* is the gene that encodes the ERα protein, while *EGR3*, *AREG* and *TFF1* are well-known estrogen-regulated genes and, thus, the main target genes of ERα signaling [56,57]. Moreover, *EGR3* has been considered as the bona fide target gene of ERα [58]. MCF-7aro cells that were plated at a cell density of 7 × 10^5^ cells/mL in six-well plates were treated with T (1 nM) with or without AIs **6**, **10a** and **13** (10 μM) over 3 days. We used as a positive control the cells treated with T plus ICI 182 780 (100 nM). In Table 1, we present the primer sequences (5′-3′) of target genes. *TUBA1A* and *ACTB* were used as housekeeping genes. The 2^−ΔΔCt^ method was applied to estimate the fold change in gene expression.

### 2.8. ER and AR Transactivation Assays

The agonistic/antagonistic activity of AIs **6**, **10a** and **13** in relation to the human AR and ER was determined as reported by our group [28,31,59], following the OECD Guidelines for the Testing of Chemicals, Tests No. 458 [60] and 455 [61], respectively.

The AR-EcoScreen cell line (RRID: CVCL_8544) (#JCRB1328, from Japanese 244 Collection of Research Bioresources Cell Bank, Tokyo, Japan), which was derived from Chinese hamster ovary (CHO-K1) cells that express the human AR with a firefly luciferase reporter construct and renilla luciferase gene for viability estimation, was applied for the AR-EcoScreen™ assay. These cells were cultured as commonly performed by our group [28,31,59]. For the experiments, the cells that were plated at cell density of 9 × 10^4^ cells/mL in 96-well white plates were incubated with AIs **6**, **10a** and **13** (0.1–10 µM) over 24 h, with or without 0.1 nM of methyltrienolone (R1881; AbMole BioScience, Houston, TX, USA), to estimate AR antagonism and agonism, respectively. Cells that were treated with R1881 (7.8 pM–1 nM) or with hydroxyflutamide (OHF; 4.1 nM–9 µM; Sigma-Aldrich Co., Saint Louis, MO, USA) were designated as positive controls for AR agonism and AR antagonism, respectively.

For the ER assay, we employed the VM7Luc4E2 cells (RRID: CVCL_6571), which were kindly supplied by Michael Denison (University of California, Berkeley, CA, USA). The cell culture conditions are the ones commonly used by our group [28,31,59]. For the experiments, VM7Luc4E2 cells were plated at 4 × 10^5^ cells/mL in 96-well white plates and treated with AIs **6**, **10a** and **13** (0.1–10 µM) over 24 h, with (ER antagonism) or without (ER agonism) 91.8 pM of E_2_ or 1 nM of T. For agonism assays, we considered as a positive control cells that were treated with T (781.2 pM–25.6 µM) or E_2_ (180 fM–367 nM), while for ER antagonism, the positive controls were cells that were treated with raloxifene (12.0 pM–24.5 nM; Biosynth Ltd., Berkshire, UK).

All data are expressed as a fold change that was set at one in relation to control cells, which are non-treated cells. Stock solutions of R1881, raloxifene, OHF, T and E_2_ were prepared in 100% DMSO (Sigma-Aldrich Co., Saint Louis, MO, USA) and stored at −20 °C. For the experiments, freshly dilutions were prepared in a cell culture medium. For all the studied conditions, the final concentrations of DMSO in the culture medium were less than 0.06%, and the controls contained this vehicle under these conditions.

### 2.9. Statistical Analysis

All data were statistical analyzed by applying the analysis of variance (ANOVA) test, with multiple comparisons being performed with Bonferroni and Tukey post-hoc tests and using GraphPad Prism 9^®^ software (Prism 9, GraphPad Software, Inc., San Diego, CA, USA). Data where the *p* value is lower than 0.05 were considered statistically significant. All the data were represented as the mean ± SEM, and all the experiments were performed in triplicate in at least three independent experiments.

## 3. Results

### 3.1. Effects on Viability of Non-Tumoral Cell Lines and of Sensitive ER+ Breast Cancer Cells

**Figure 2:**
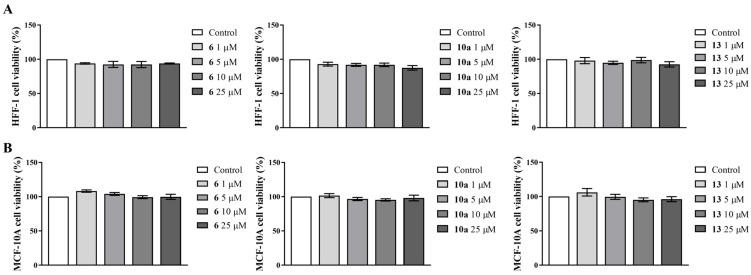
Effects of AIs **6**, **10a** and **13** on the cell viability of the non-tumor cell lines HFF-1 (**A**) and MCF-10A (**B**). HFF-1 and MCF-10A cells were incubated with each AI (1–25 μM) over 6 days. Untreated cells representing 100% of cell viability were designated as controls, with the effects of AIs being normalized to these control values.

To estimate the potential cytotoxicity of the new AIs **6**, **10a** and **13** on non-tumor cells, their effects on the cell viability of two different human non-tumoral cell models—the fibroblastic cell line (HFF-1) and the epithelial breast cell line (MCF-10A)—were investigated by using MTT assays after 6 days of treatment. The results demonstrated that the new compounds had no impact on the viability of both non-tumor cell models (Figure 2).

**Figure 3:**
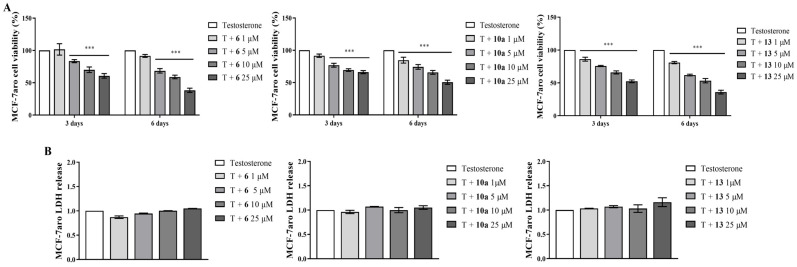
Effects of AIs **6**, **10a** and **13** on the viability of MCF-7aro cells. Cells were treated with T (1 nM) and each AI (1–25 μM) for 3 and 6 days. The actions of each AI were evaluated by MTT (**A**) and LDH assays (**B**). Untreated cells representing 100% of cell viability and 1 unit value of LDH release were designated as controls, with data of AIs treatment being normalized to these control values. *** (*p* < 0.001) denote statistically significant differences between control cells and AI-treated cells.

Considering their non-cytotoxic profile in non-tumor cells, the behavior of the new AIs on MCF-7aro cell viability was further investigated by MTT and LDH experiments, after each incubation time point studied. As presented in Figure 3A, all the new AIs caused a significant (*p* < 0.001) and dose- and time-dependent reduction of MCF-7aro cell viability. Comparing all the compounds, AI **13** was the most efficient, since its ability to decrease cell viability was verified for all the concentrations and times of treatment. Considering LDH release from the cytosol into the medium, no significant variations were observed (Figure 3B), indicating that our compounds did not induce cell membrane disruption.

### 3.2. Behavior of AIs on Proliferation and on Cell Death of Sensitive Breast Cancer Cells

**Figure 4:**
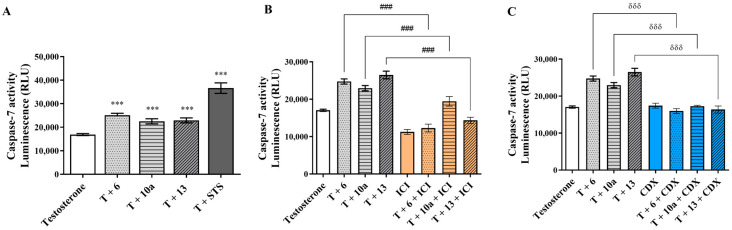
Effects of AIs **6**, **10a** and **13** on MCF-7aro cell death. Cells were incubated with T (1 nM) and with or without each AI at 10 μM for 3 days, in the presence or absence of CDX (1 μM) or ICI (100 nM). Cells without AI treatment were designated as controls, while as a positive control we considered cells incubated with T plus STS (10 µM). The effects on apoptotic cell death were determined by the activation of the effector caspase-7 (**A**), and data are presented in relative luminescence units (RLUs). The involvement of ERα (**B**) and AR (**C**) in the promotion of apoptosis was assessed by caspase-7 activity after incubation of AIs with ICI or CDX, respectively. *** (*p* < 0.001) denotes statistically significant differences between control cells and AI-treated cells, δδδ (*p* < 0.001) indicates differences between AI-treated cells in the presence or absence of CDX and ### (*p* < 0.001) the differences between AI-treated cells in the presence or absence of ICI.

To further understand whether the impact on cell viability was associated with anti-proliferative or cell death effects, the actions of the new AIs on cell cycle progression and caspase-7 activation were explored in sensitive MCF-7aro cells. The results presented in Table 2 and in Supplementary Figure S1 show that all the new AIs significantly impaired cell cycle progression by arresting cells in the G_0_/G_1_ phase (*p* < 0.001), which, consequently, reduced the number of cells in the S phase (*p* < 0.001) when compared to the control (T-treated cells). In addition, all AIs caused a significant (*p* < 0.001) activation of the effector caspase-7 (Figure 4A). As predicted, the positive control STS also significantly (*p* < 0.001) increased the activity of this caspase.

### 3.3. The Underlying Molecular Targets of the New AIs in Sensitive Breast Cancer Cells

To understand the mechanism behind the anti-growth effects exerted by the new AIs, their actions on three main targets—aromatase, ER and/or AR—were investigated.

**Figure 5:**
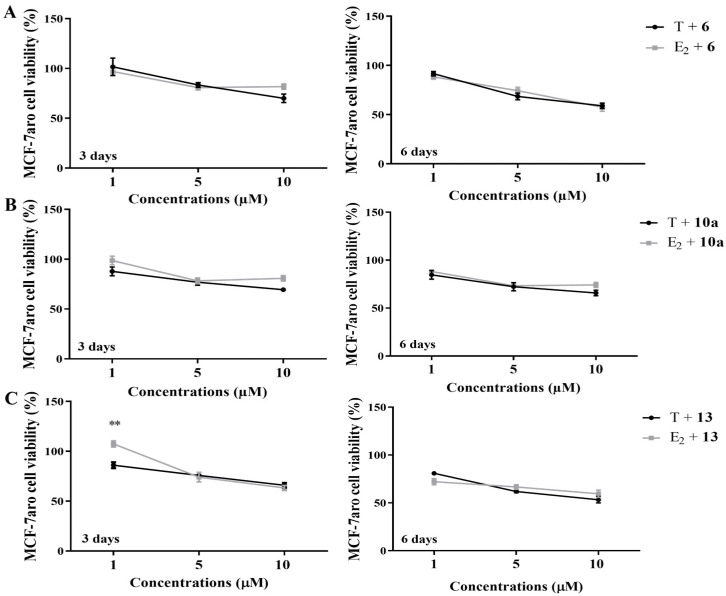
The impact of aromatase on the effects exerted by AIs **6** (**A**), **10a** (**B**) and **13** (**C**) on ER^+^ breast cancer cells, evaluated by the MTT assay. MCF-7aro cells were incubated with AIs (1, 5 and 10 μM) plus T (1 nM) or E_2_ (1 nM) over 3 and 6 days. Untreated cells representing 100% of cell viability were designated as control, being data of AIs normalized to these control values. ** (*p* < 0.01) indicate significant differences between AI-treated cells incubated with E_2_ or with T.

We previously reported that AIs **6** [39], **10a** [40] and **13** [40] inhibit aromatase activity in more than 90% in two different aromatase-enriched models (human placental microsomes and MCF-7aro cells). Considering this, we studied if their effects on cells were influenced by their strong ability to inhibit aromatase, by comparing the impact on MCF-7aro cell viability when AI-treated cells are stimulated with the aromatase substrate (T) or product (E_2_). The results presented in Figure 5 demonstrate that, except for AI **13** at the lowest concentration (1 µM), no differences were detected between AI-treated cells incubated with T or E_2_.

**Figure 6:**
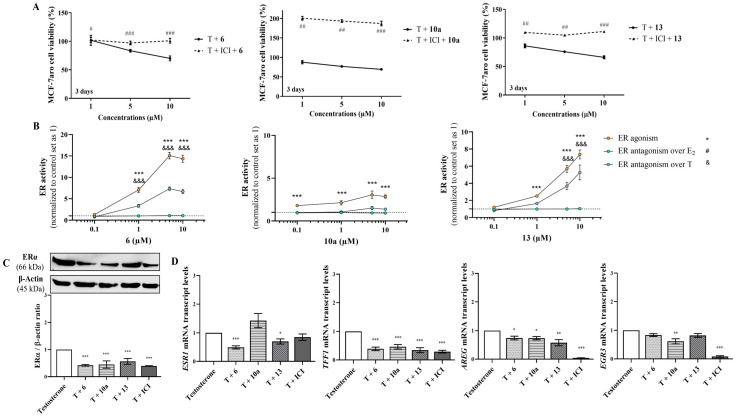
The impact of ERα on the effects exerted by AIs **6**, **10a** and **13**. (**A**) MCF-7aro cell viability effects were determined by MTT assay after treatment over 3 days with T (1 nM) plus AIs (1, 5 and 10 μM) in the presence or absence of ICI (100 nM). (**B**) ER transactivation assays to explore the effects on ER activation, using VM7Luc4E2 cells incubated with AIs (0.1–10 μM), with (ER antagonism) or without (ER agonism) the hormones T or E_2_. (**C**) Effects of AIs **6**, **10a** and **13** (10 μM) on ERα protein expression. β-actin was used as a loading control, being data of densitometry represented as ERα/β-actin ratio. (**D**) Effects of AIs **6**, **10a** and **13** (10 μM) on mRNA transcription of *ESR1*, *TFF1*, *AREG* and *EGR3* genes in MCF-7aro cells. The mRNA transcript levels of treated cells were quantified using the housekeeping gene *ACTB*. Cells without treatment were used as control, to which all results in AI-treated cells were normalized. Cells treated with T plus ICI (100 nM) represented positive control. # (*p* < 0.05), ## (*p* < 0.01) and ### (*p* < 0.001) denote differences in MCF-7aro cells treated with AIs in the absence or presence of ICI, while * (*p* < 0.05), ** (*p* < 0.01), and *** (*p* < 0.001) denote differences of AI-treated cells in relation to control cells (T). On the other hand, the differences of AI-treated cells in contrast to control and in relation to ER agonism are indicated by *** (*p* < 0.001), while in relation to ER antagonism over T are expressed by &&& (*p* < 0.001).

To explore the influence of ER on the effects exerted by AIs, sensitive cells were incubated with the compounds in the presence or absence of the selective ER degrader (SERD) ICI 182780 (Fulvestrant). Interestingly, all the AIs induced significant differences (*p* < 0.05; *p* < 0.01; *p* < 0.001) in the presence or absence of ICI, as a reduction of cell viability was induced only in cells without ICI (Figure 6A). As these results suggest the involvement of ER, we further used another approach to explore ER modulation by performing ER transactivation assays using the VM7Luc4E2 cells incubated with AIs, with (ER antagonism) or without (ER agonism) the hormones E_2_ or T. As presented in Figure 6B, ER transactivation assays showed that all the AIs presented ER agonistic properties (*p* < 0.001), except for the lowest concentration of AIs **6** and **13**, with AI **10a** being the least effective. In the presence of E_2_ or T, no antagonistic activity was exerted by the AIs. In addition, no effect was induced on VM7Luc4E2 cell viability under any of the tested conditions (Supplementary Figure S2A). To better understand these observations, the effects of AIs on ERα protein and *ESR1* expression, as well as on the transcription of the ER-regulated genes (*TFF1*, *AREG* and *EGR3*) related to the activation of ER signaling, were investigated on T-stimulated MCF-7aro cells. The results presented in Figure 6C demonstrate that all the AIs significantly reduced (*p* < 0.001) the ERα protein expression levels in comparison to the control—a behavior also displayed by ICI (Supplementary Figure S3A,B). Moreover, except for AI **10a,** which caused no effect, the other AIs also significantly decreased (*p* < 0.05, *p* < 0.001) the mRNA transcription of the *ESR1* gene (Figure 6D). Curiously, and in relation to the control, only AI **10a** triggered a significant (*p* < 0.01) reduction in the mRNA transcription of the *EGR3* gene, whereas all AIs significantly decreased (*p* < 0.05, *p* < 0.01, *p* < 0.001) *TFF1* and *AREG* genes (Figure 6D). ICI prevented the activation of all these genes. Additionally, to better understand the role of ER, the promotion of apoptosis was explored in AI-treated cells in the presence of ICI. Interestingly, under these conditions, ER blockage impaired the activation of caspase-7, since significant (*p* < 0.001) differences between AI-treated cells in the presence or absence of ICI were detected (Figure 4B).

**Figure 7:**
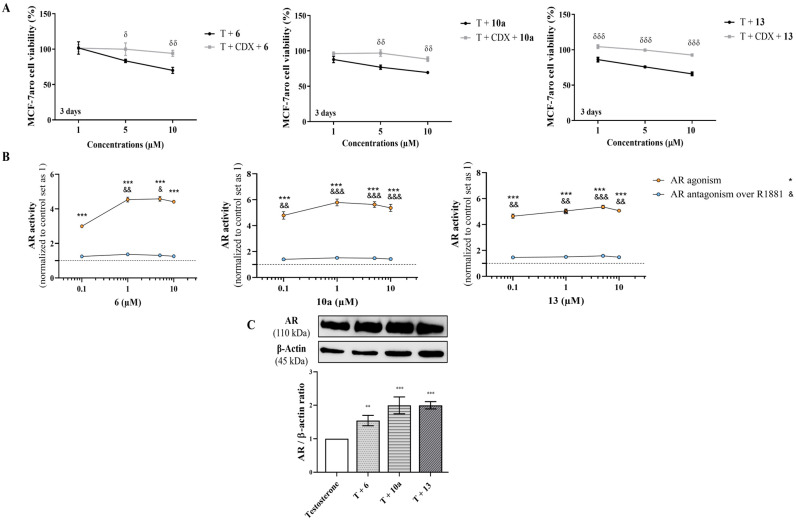
The impact of AR on the effects exerted by AIs **6**, **10a** and **13**. (**A**) MCF-7aro cell viability effects were determined by MTT assay after treatment, over 3 days, with T (1 nM) plus AIs (1, 5 and 10 μM) in the presence or absence of CDX (1 µM). (**B**) The AR transactivation assay, AR-EcoScreen™, was used to explore the effects of AIs (0.1–10 μM) with (AR antagonism) or without (AR agonism) R1881 (0.1 nM). (**C**) Effects of AIs (10 μM) on AR protein expression. β-actin was used as a loading control, being data of densitometry represented as AR/β-actin ratio. Cells without AIs treatment were used as control, to which all results in AI-treated cells were normalized. δ (*p* < 0.05), δδ (*p* < 0.01) and δδδ (*p* < 0.001) denote differences of MCF-7aro cells incubated with AIs in the presence or absence of CDX, while ** (*p* < 0.01) and *** (*p* < 0.001) denote differences of AI-treated cells in relation to control cells. On the other hand, the differences of AI-treated cells in contrast to control and in relation to AR agonism, are presented by *** (*p* < 0.001), whereas in relation to AR antagonism are indicated as & (*p* < 0.05), && (*p* < 0.001) and &&& (*p* < 0.001).

In addition, to explore the role of AR, we compared the behavior of AI-treated sensitive cells with or without the AR antagonist CDX, as well as their effects on AR protein levels and the agonistic/antagonistic properties of each compound (AR transactivation assay). Figure 7A shows that when AR is blocked by CDX, none of the AIs reduced cell viability, which suggests the involvement of AR. In fact, AR transactivation assays performed with the AR-EcoScreen™ assay with (AR antagonism) or without (AR agonism) R1881 proved that all the AIs presented significant (*p* < 0.001) AR agonistic properties and, at same time, no antagonistic activity (Figure 7B), without any significant effect on cell viability (Supplementary Figure S2B). In addition, as presented in Figure 7C, all AIs have the ability to significantly (*p* < 0.01, *p* < 0.001) increase the AR protein levels, in contrast to the control (Supplementary Figure S3C,D). Interestingly, the presence of CDX impaired the activation of caspase-7, and we detected differences (*p* < 0.001) in cells treated with AIs in the presence or absence of CDX (Figure 4C).

### 3.4. Effects on Resistant ER^+^ Breast Cancer Cells

**Figure 8:**
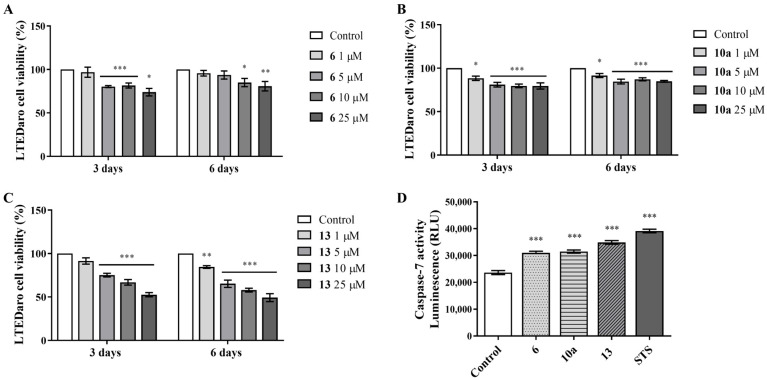
Effects of **6**, **10a** and **13** on AI-resistant ER+ breast cancer cells, LTEDaro. The effects of AIs **6** (**A**), **10a** (**B**) and **13** (**C**) (1–25 μM) on cell viability were determined by the MTT assay after 3 and 6 days. (**D**) Effects of AIs (10 μM) on cell death after 3 days, by assessing caspase-7 activity. Cells treated only with STS (10 µM) were denominated as positive control. Data are expressed as relative luminescence units (RLUs). Untreated cells were designated as control, to which all results of AI-treated cells were normalized. * (*p* < 0.05), ** (*p* < 0.01) and *** (*p* < 0.001) indicate differences between the control and AI-treated cells.

The ability of AIs **6**, **10a** and **13** to affect resistant stages of the disease and re-sensitize LTEDaro cells was investigated by exploring the effects of new molecules on cell viability (MTT assay) and in the promotion of apoptosis, through the analysis of caspase-7 activation. The results presented in Figure 8A–C demonstrate that all the AIs significantly reduced (*p* < 0.05, *p* < 0.01, *p* < 0.001) LTEDaro viability, except at the lowest concentration of AI **6**, at both incubation times, and of AI **13** for 3 days. Furthermore, all AIs augmented (*p* < 0.001) the activity of the effector caspase-7 (Figure 8D).

## 4. Discussion and Conclusions

It is currently known that endocrine therapies applied in the treatment of ER+ breast cancer, such as AIs and anti-estrogens, have demonstrated limitations, mainly due to the emergence of resistance. This has led to the implementation of other strategies, such as their combination with drugs directed towards other molecular targets [3,6,8,62] or the use of novel SERD molecules [8,9,10,14]. However, some of these therapies still show poor efficacy, some toxicity and/or even tumor recurrence [3,11,13,62]. Therefore, the development of novel drugs with different chemical entities or modes of action, as well as a direct or allosteric modulation of key cellular targets or the blockade of key signaling pathways involved in cell proliferation, is essential for targeted molecular therapy in order to increase efficacy, reduce toxicity and/or minimize recurrence [62]. Recently, multi-target/polypharmacological molecules are arising, which are based on the concept that a single molecule can interact with two or more targets simultaneously. These molecules are often more potent, have higher efficacy, have a better safety profile, exhibit a superior pharmacokinetic profile, and are associated with reduced risks of drug interactions [1,15,16,17,18,20]. Furthermore, it has been highlighted that additive therapeutic effects may be achieved when the targets belong to the same or functionally complementary pathways [15,17,19]. Indeed, our group has been looking for this type of multi-target/polypharmacological compounds, and we have already discovered different steroidal [28,33,34,35] and non-steroidal molecules [30,31,32] that simultaneously target AR, ER and aromatase in order to impair the growth of cancer cells.

Recently, we found that three new steroidal compounds (**6**, **10a** and **13)**, designed and synthesized by our group, strongly inhibit aromatase enzyme either in human placental microsomes or in breast cancer cells [39,40]. Compound **6** presents an IC_50_ value of aromatase inhibition of 0.405 µM [40], compound **10a** has an IC_50_ value of 0.27 µM [40] and compound **13** has an IC_50_ value of 0.25 µM [39]. To follow up on this work and in an attempt to find new multi-target drugs, we investigated the anti-cancer potential of these three new AIs on sensitive (MCF-7aro) and resistant (LTEDaro) ER^+^ breast cancer cells, as well as their ability to also target ER and/or AR. Our results demonstrated that AIs **6**, **10a** and **13** caused a non-cytotoxic profile in two different non-tumoral cell models—HFF-1 and MCF-10A cells—and thus can be considered safe compounds. Moreover, they showed the ability to impair the growth of sensitive breast cancer cells by significantly reducing MCF-7aro viability and disrupting cell cycle progression at the G_0_/G_1_ phase. These anti-cancer properties were accompanied by the occurrence of apoptosis through the activation of the effector caspase-7, with no cytotoxic profile in relation to cell membrane disruption, as no effect on LDH release was observed. Similar behaviors on MCF-7aro cell proliferation and death were already identified by our group for other A-, B- and D- ring modified steroids [28,33,34,35,54,63,64]. Moreover, it is known that the AIs in clinical use (Exe, Let and Ana) also arrest the cell cycle and trigger apoptosis in this cell model [44,47,48,65]. Besides this, interestingly, the new molecules can re-sensitize the resistant cell model. In fact, all the new compounds reduced LTEDaro cell viability by activating caspase-7, an effector caspase of the apoptotic pathway. Some of our previously synthesized AIs with modifications on the A-, B- and D-rings also exhibit this behavior in LTEDaro cells [28,33,35,63]. Interestingly, it was the compound with only C-ring modifications, AI **13**, that showed the most pronounced effects on the proliferation of sensitive and resistant cells, which indicates that these modifications may help in the drug design of future novel molecules.

In order to unveil the multi-target potential of these promising new molecules and identify their mechanism of action, we investigated whether their impact on MCF-7aro cells were influenced by aromatase, ER and/or AR—the key therapeutical targets for this type of cancer. It should be pointed out that ERα, through the action of estrogens, is the receptor liable for the growth/survival of ER+ breast tumors, while AR acts as an oncogenic or tumor suppressor, depending on the endocrine status of ER+ cancer cells [1,2,3,21,24,25,26,27]. We previously reported that AIs **6** [39], **10a** [40] and **13** [40] presented strong anti-aromatase activity—of 89.53%, 90.77% and 96.39%, respectively—in MCF-7aro cells. However, our studies in these cells stimulated with T, the aromatase substrate, or E_2_, the aromatase product, suggested that their in vitro effects are independent of aromatase inhibition. We previously reported a similar mechanism of action in relation to aromatase for other compounds with modifications in the A-, B- and D-rings [33,34,35], which, instead, exerted effects dependent on ERs and/or AR. In fact, our results suggest that the effects of compounds **6**, **10a** and **13**, apart from a strong inhibition of aromatase, also are dependent on ERα and AR. Indeed, when ERα is blocked with the SERD ICI 182780 in AI-treated MCF-aro cells, no reduction of MCF-7aro cell viability was observed. Considering this, we further explored the ER agonistic/antagonistic properties of these molecules. The ER transactivation assays suggest that AIs **6** and **13** display ER agonism actions, while AI **10a** may exert partial ER agonism only in the absence of hormones—a behavior that is abrogated in the presence of hormones. Nevertheless, it should be noted that some molecules being considered as SERMs may display either agonist and/or antagonist activity, depending on tissue specificity [61,66]. In addition, their effects on ER and ERα signaling activation were explored. Interestingly, our results showed that all the new AIs induced a decrease in ERα expression and that only AIs **6** and **13** reduced the transcript levels of the *ESR1* gene. Furthermore, the transcription of the genes that are ERα-regulated (*AREG* and *TFF1*) was prevented by all AIs, though only AI **10a** reduced the levels of the *EGR3* gene—the most specific target gene of ERα [58]. Collectively, these results indicate the involvement of ERα on AIs actions in ER+ breast cancer cells. Curiously, AI **10a** presented a behavior like the SERD ICI 182780 and the new SERD AZD9496 [67], indicating that it may be considered as a potential ER degrader, although more studies should be carried out to support this. Recently, it was highlighted that SERDs degrade ER through the differential recruitment of the E3 ubiquitin protein ligases UBR5 or RNF111, which may affect agonism and antagonism activity [68]—a potential explanation for the ER degrader mechanism in MCF-7aro cells and the agonistic activity observed. However, in relation to ERα-regulated genes, the mechanism of action on ER signaling induced by AI **10a** is similar to the non-steroidal AIs Let and Ana [48], with the advantage that AI **10a** down-regulates ER protein levels, while Ana and Let increase ER gene and protein expression, probably as a compensation mechanism of impaired ER signaling [48]. On the other hand, the behaviors of AIs **6** and **13** pointed them out as potential ER modulators, with their mechanism of action in relation to the *EGR3* gene being similar to the one reported for Exe [48], but with the advantage that these new AIs reduce ER protein levels and synthesis. These mechanisms of action highlight their anti-cancer potential, since by modulating ER levels, synthesis and signaling, they are also considered ER modulators and thus multi-target drugs, which is a potential therapeutic advantage. Another observation corroborating the importance of targeting ER was that the occurrence of apoptosis was prevented when ERα was blocked with the SERD ICI 182780 in AI-treated MCF-aro cells. Recently, we also described a similar mechanism of action in relation to ERα for Oxymestane-D1, a derivative of Exe [28], and for the non-steroidal compound designated as tamoxifen bisphenol [30]—molecules that are also considered multi-target drugs. In addition, ER-dependent effects in MCF-7aro cells were also reported for other A-, B- and D-ring modified steroids [33,34].

Regarding AR, cell viability impacts in the absence or presence of the AR antagonist CDX demonstrated that an AR blockade impaired the loss of viability exerted by AIs, suggesting an AR dependence. In fact, transactivation assays performed with an AR-EcoScreen™ assay proved that all the new AIs presented AR agonistic properties. Additionally, these molecules are able to induce AR overexpression. As 80–95% of breast ER+ tumors overexpress AR [22,23], and depending on endocrine status and treatment applied, the AR may present an oncogenic or tumor suppressor role [2,3,24,25,26,27,48], the impact of AR on AIs actions was explored. Cell death studies revealed that when AR is impaired by CDX, the induction of apoptosis was prevented. This evidence indicates that AR presents a pro-cell death role and that these new AIs up-regulate AR levels and act as AR agonists in order to induce cell death, which is also a therapeutic benefit. This AR-dependent biological effect is an advantage over the reference steroidal AI Exe, since for Exe this receptor plays a pro-survival role [25,48]. On the other hand, this mechanism is similar to those induced by the reference AIs Ana and Let [48] and Oxymestane-D1 [28]. Interestingly, these results, together with previous ones [28,33,34], also highlighted that synthesized steroidal derivatives of androstenedione with modifications in the A-, B- and D-rings may present an AR-dependent effect on MCF-7aro cells to promote cell death. This could be a consequence of aromatase inhibition and the subsequent increase in androgen levels, since all our studied AIs with these chemical structure modifications present this behavior.

We previously reported that independently of the chemical A-ring and B-ring substitutions, the presence of a carbonyl group at C-17 in the D-ring of the steroidal scaffold is relevant for an AR-dependent effect. On the contrary, the presence of a hydroxyl group at the C-17 position in the D-ring is supposed to be associated to ER-dependent effects [33]. More recently, our group highlighted that, at C-17, the existence of a carbonyl group is also relevant for an AR- and ER-dependent effect [34], which suggest that, at the C-17 position, either the hydroxyl or the carbonyl group may be a determinant for an ER-dependent effect. Looking at the chemical structure of the new AIs, it seems likely that a carbonyl group in the C-17 position in the steroidal scaffold is relevant for an AR-dependent effect and an ER-dependent mechanism. Nevertheless, this type of SAR study did not allow us to infer which functional groups on steroidal scaffolds are associated with ER-dependent mechanisms, so this relationship is still unclear.

Overall, these results emphasize the discovery of new steroidal compounds with multi-target action that simultaneously target aromatase, ER and AR, by acting as AIs, ER modulators and AR agonists and promote apoptosis in breast cancer cells. These multi-target actions are clinically beneficial for ER+ breast cancer, as all these targets play crucial roles in the proliferation, survival and activation of the programmed cell death of luminal A breast cancers. Furthermore, these new steroidal compounds re-sensitize resistant cells by promoting cell death, which is also a therapeutic benefit over the reference AIs used in the clinic. Therefore, we believe that this study is a breakthrough on drug discovery as it allows the finding of new anti-cancer molecules with appealing anti-cancer and multi-target properties for the Luminal A subtype of ER+ breast cancer, although in vivo studies should be performed to translate the findings for a clinical setting.

## Figures and Tables

**Table 1:** qPCR conditions and primer sequences for housekeeping and target genes.

Target Gene	Primer Sequences (5′–3′)		Ta/°C
	Sense	Anti-Sense	
*TUBA1A*	CTGGAGCACTCTGATTGT	ATAAGGCGGTTAAGGTTAGT	55
*ACTB*	TGCCATCCTAAAAGCCACCC	AGACCAAAAGCCTTCATACATCTC	55
*ESR1*	CCTGATCATGGAGGGTCAAA	TGGGCTTACTGACCAACCTG	55
*TFF1*	GTGGTTTTCCTGGTGTCACG	AGGATAGAAGCACCAGGGGA	55
*EGR3*	GACTCCCCTTCCAACTGGTG	GGATACATGGCCTCCACGTC	56
*AREG*	TGTCGCTCTTGATACTCGGC	ATGGTTCACGCTTCCCAGAG	56

**Table 2:** Effects of compounds **6**, **10a** and **13** on MCF-7aro cell cycle progression.

	G_0_/G_1_	S	G_2_/M
Testosterone	73.77 ± 0.74	7.84 ± 0.29	16.00 ± 0.73
T + **6**	82.19 ± 0.53 ***	2.67 ± 0.17 ***	14.26 ± 0.41 *
T + **10a**	85.92 ± 1.23 ***	2.00 ± 0.19 ***	11.97 ± 0.85 ***
T + **13**	84.50 ± 0.67 ***	2.16 ± 0.22 ***	12.16 ± 0.33 ***

Cells stimulated with T (1 nM) were treated with **6**, **10a** and **13** at 10 μM over 3 days, being after the treatment, stained with PI (1 μg/mL) and further assessed by flow cytometry. * (*p* < 0.01) and *** (*p* < 0.001) denote significant differences between the treated cells and control.

## Data Availability

All data supporting the findings of this study are available within the paper and on Supplementary Figures, although it can be shared on request.

## Supplementary Materials

The following supporting information can be downloaded at: https://www.mdpi.com/article/10.3390/cancers17020165/s1, Figure S1: Effects of compounds **6**, **10a** and **13** on MCF-7aro cell cycle distribution. Figure S2: Effects of compounds **6**, **10a** and **13** on viability of VM7LucE2 cells (A) and CHO-K1 cells (B). Figure S3: The original Western blot images for the Western blots presented in the manuscript can be found.
