# Supplementary material for: New Promising Steroidal Aromatase Inhibitors with Multi-Target Action on Estrogen and Androgen Receptors for Breast Cancer Treatment
Source: Cancers (Basel). 2025 Jan 7;17(2):165. doi: 10.3390/cancers17020165 (PMC11763961; doi:10.3390/cancers17020165)
Supplement: Supplementary file 1 [file cancers-17-00165-s001.zip › cancers-3350541-supplementary.pdf]

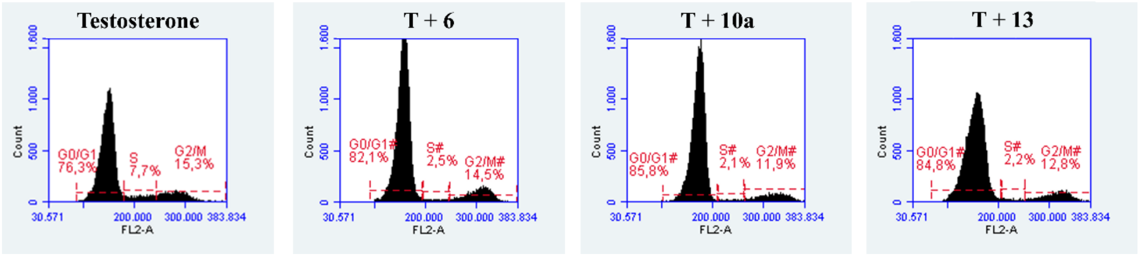

Supplementary Figure S1: Effects of compounds **6**, **10a** and **13** on MCF-7aro cell cycle distribution. Cells were treated with AIs **6**, **10a** and **13** (10 µM) during 3 days and subjected to flow cytometric analysis after PI staining. Cells cultured with testosterone (T) (1 nM) represent the maximum of cell proliferation and were considered as control. Data presented in histograms were analysed with BD Accuri™ C6 analysis software and are representative of one independent assay.

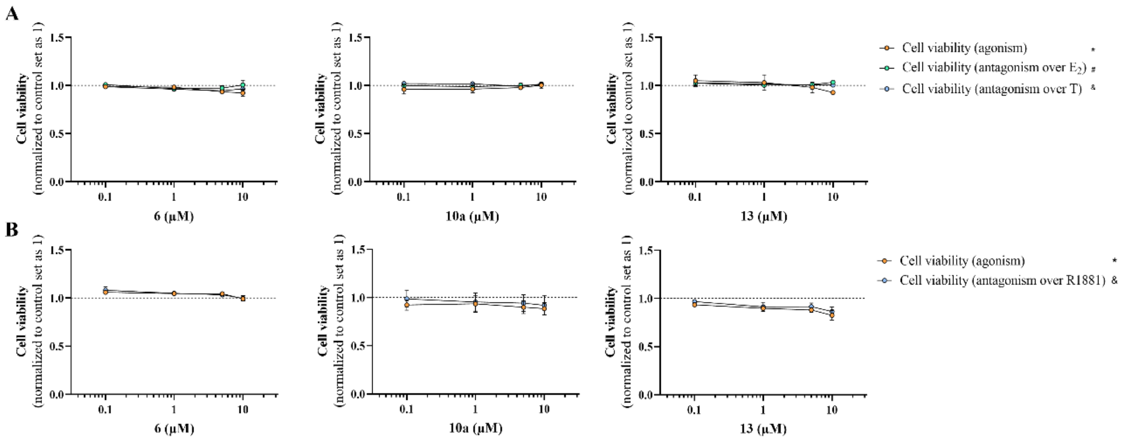

Supplementary Figure S2: Effects of compounds **6**, **10a** and **13** on viability of VM7LucE2 cells (**A**) and CHO-K1 cells (**B**). VM7LucE2 cells were treated with AIs **6**, **10a** and **13** (0.1 – 10 µM) with (ER antagonism) or without (ER agonism) testosterone (T) or estradiol (E2), during 24h. CHO-K1 cells were treated with AIs **6**, **10a** and **13** (0.1 – 10 µM) with (AR antagonism) or without (AR agonism) R1881 (0.1 nM) for 24h. Cells only cultured with medium were considered as control, being the effects of AIs normalized considering these control values.

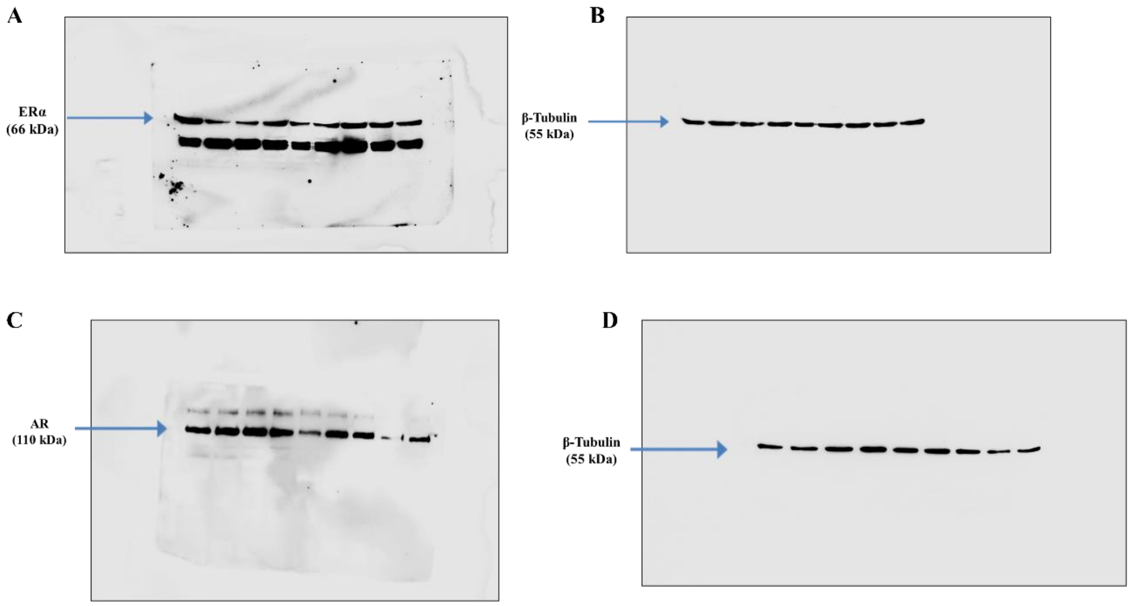

Supplementary Figure S3: Original Western Blot images of the Western-Blots presented in the manuscript. All the experiments were performed using β-actin as a loading control. **(A)** Western Blot of ERα shown on figure 6C. The order of the samples in the gel was T, T + **6** (10μM),T+ **10a** (10μM),T+**13** (10μM),T+ICI, X, X, X, X. **(B)** Western Blot of β-actin shown on figure 6C. The order of the samples in the gel was T, T + **6** (10μM),T + **10a** (10μM),T+**13** (10μM),T+ICI, X, X, X, X. **(C)** Western Blot of AR shown on figure 7C. The order of the samples in the gel was T, T + **6** (10μM),T+ **10a** (10μM),T + **13** (10 μM), X, X, X, X, X. **(D)** Western Blot of β-actin shown on figure 7C. The order of the samples in the gel was T, T + **6** (10μM),T+ **10a** (10μM),T+**13** (10μM),X, X, X, X, X. X represents samples of unrelated experiments.
